# A Recipe for ORANGE-CAKE—This Time with Two Layers!

**DOI:** 10.1523/ENEURO.0311-22.2022

**Published:** 2022-08-23

**Authors:** Keely Duff

**Affiliations:** Department of Biology, University of South Carolina, Columbia, South Carolina 29208

**Keywords:** CRISPR/Cas9, fluorescence microscopy, knock-in, multiplex genome editing, neurons

As readers of *eNeuro*, we would agree that neurons are important cells—you are using them right now to read and understand this article [unless you are a sentient AI (artificial intelligence)–LaMDA (Language Model for Dialogue Applications); I would love to be friends!]. There are still a lot of unknowns about how neuronal proteins contribute to neuronal function. What exactly are the proteins in your neurons doing as you are reading this? We may not be able to determine that yet, but being able to visualize proteins in living neurons in culture is an important step toward that goal. The benefit of being able to “see” proteins is that you can determine where they go and how they move around in living cells. You can also get an idea of which proteins are interacting with each other.

Fluorescent labeling of proteins has proven to be a useful tool for visualizing proteins in living cells. Historically, though, this method has had its drawbacks. In order to add a fluorescent tag to a protein, a gene encoding the tag and the protein of interest would be overexpressed in cells (typically done with a plasmid or virus). This can provide valuable information but can also result in atypical cellular function as it will alter normal protein levels.

It would be great to be able to tag the existing gene without altering expression levels (i.e., at the endogenous level) as this would prevent that problem. This is especially complicated in neurons because they are postmitotic, making it impossible to isolate and expand clones and precluding multiple independent rounds of gene modification.

Labeling individual endogenous proteins in neurons has been successfully accomplished using CRISPR/Cas9-mediated techniques. CRISPR works by coexpressing an endonuclease (e.g., Cas proteins like Cas9) and a guide RNA (gRNA) homologous to the target gene. Cas9 and the gRNA form a ribonucleoprotein complex, which then creates a double-stranded break (DSB) within the target DNA. This DSB is repaired using your “donor” DNA construct (expressed separately from Cas9 and gRNA, and carrying the fluorescent tag) and the endogenous DNA repair machinery of the cell.

Multiplex labeling (labeling more than one type of protein simultaneously) remains challenging in neurons; this is largely because of the way they prefer to repair their DSBs. One general repair method available is the homology-directed repair pathway (HDR). The HDR uses a repair template with high homology to the target sequence, which lends this method high fidelity; however, its efficacy in neurons is too low for routine use. The repair pathway favored by neurons and other postmitotic cells is nonhomologous end joining (NHEJ); it requires homology between your donor DNA and the target locus, and the donor DNA can indiscriminately integrate into any available DSB. Editing two genes requires two DSBs—one in each gene—and thus the lack of homology-driven integration of the donor strongly increases off-target labeling (one or both donors inserting into the wrong DSB cross talk).

The laboratory of Dr. Harold D. MacGillavry (Utrecht University, Utrecht, the Netherlands) previously developed a recipe book for performing CRISPR/Cas9 labeling of endogenous proteins in neurons: Open Resources for the Application of Neuronal Genome Editing (ORANGE; Willems et al., 2020). This toolkit, including their cloning vector and ∼50 knock-in constructs is available from Addgene. ORANGE was used to develop a strategy called Conditional Activation of Knock-in Expression (CAKE). The laboratory used ORANGE-CAKE to label endogenous proteins and perform live-cell super-resolution microscopy in different model systems [dissociated neurons, organotypic slice cultures, and *in vivo* (Cas9 transgenic mouse line)]. The method was successful with several genes and gene pairs, but cross talk between loci in some of those combinations was observed.

What the laboratories of Dr. Arthur P. H. de Jong (Utrecht University, Utrecht, the Netherlands) and Dr. MacGillavry brought to the table here (Droogers et al., 2022; Fig. 1) is a way to essentially eliminate that cross talk between donor DNAs and to enable multiplex gene editing. The trick was to separate the genome editing events in time. While both knock-in vectors are delivered simultaneously, only one of them is active, leading to exclusive editing of the first gene. After a few days, the activation of Cre recombinase switches off the first vector, and switches on the second vector, exclusively editing the second gene. This sequential editing of genes essentially eliminates cross talk.

**Figure 1.**
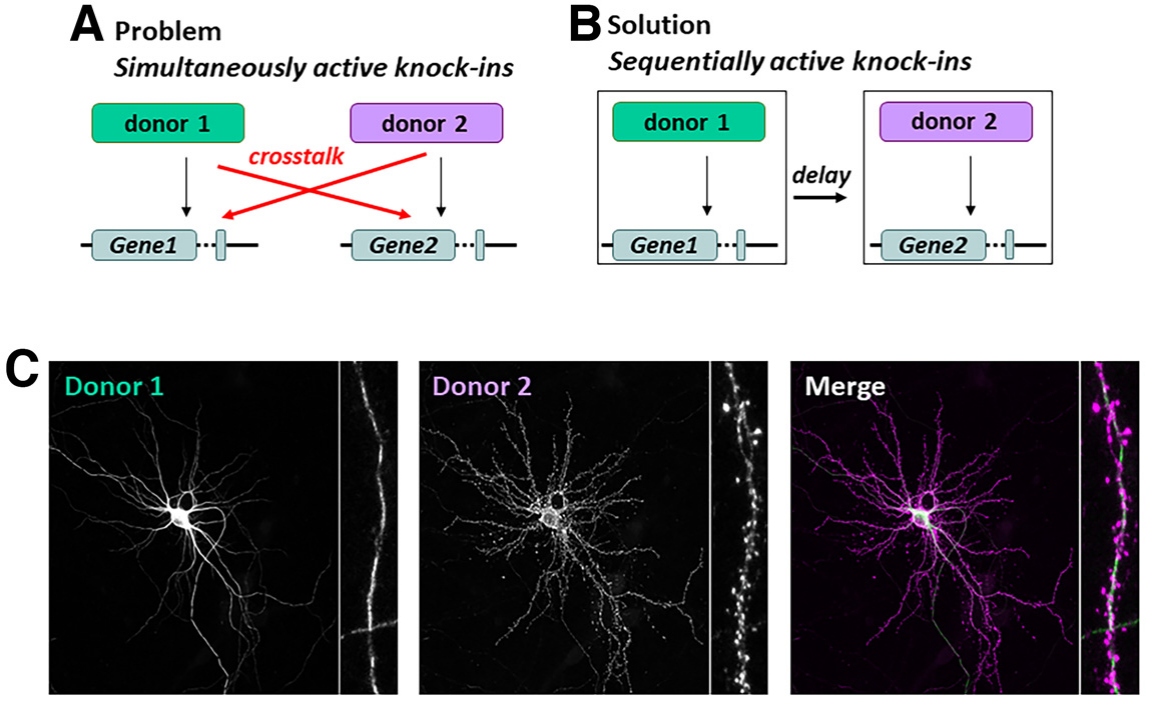
***A***, Diagram of the problem facing multiplex knock-in strategies using NHEJ. With simultaneous editing of multiple genes, donor DNAs could be integrated in either allele, leading to cross talk. ***B***, CAKE is designed to introduce and control a delay between two genome-editing events, so that cross talk can be avoided. ***C***, Example confocal image of a successful multiplex knock-in using CAKE.

In the 2022 article (Droogers et al., 2022), the de Jong laboratory lays out the framework of how they optimized ORANGE-CAKE across different gene combinations and knock-in strategies. They also introduced an exciting new application by using their ORANGE-CAKE to add inducible dimerization modules (in addition to the fluorophores) to control the positioning of endogenous proteins. An inducible dimerization module consists of a pair of molecular tags added to each protein of interest (chemically, by each binding one side of a small ligand; or optogenetically using light). In the article, they used a chemical module to link AMPA receptors to PSD-95, a postsynaptic scaffolding protein to anchor the receptors to the synapse.

This unlocks a huge world of potential to precisely and acutely manipulate the positioning of not just receptors, but also other molecules and organelles. Droogers et al. (2022) also suggest that many different modules can be introduced using this method. For example, CAKE could also be applied in combination with Förster resonance energy transfer reporters, bimolecular fluorescence complementation (Tebo and Gautier, 2019), or proximity biotinylation assays (De Munter et al., 2017).

In summary, this two-layer CAKE system has broad applications for multiplex imaging of various endogenous proteins, while ORANGE-CAKE offers a useful tool for a broad range of applications in neurons. Let’s get baking.

## Synthesis

Reviewing Editor: Deanna Smith, University of South Carolina
